# Implication of Sphingolipid Metabolism Gene Dysregulation and Cardiac Sphingosine-1-Phosphate Accumulation in Heart Failure

**DOI:** 10.3390/biomedicines10010135

**Published:** 2022-01-08

**Authors:** Lorena Pérez-Carrillo, Isaac Giménez-Escamilla, Luis Martínez-Dolz, Ignacio José Sánchez-Lázaro, Manuel Portolés, Esther Roselló-Lletí, Estefanía Tarazón

**Affiliations:** 1 Myocardial Dysfunction and Cardiac Transplantation Unit, Health Research Institute Hospital La Fe (IIS La Fe), Avd. Fernando Abril Martorell 106, 46026 Valencia, Spain; lorena_perezc@iislafe.es (L.P.-C.); igies@alumni.uv.es (I.G.-E.); martinez_luidol@gva.es (L.M.-D.); ignaciosanchezlazaro@gmail.com (I.J.S.-L.); portoles_man@gva.es (M.P.); 2 Center for Biomedical Research Network on Cardiovascular Diseases (CIBERCV), Avd. Monforte de Lemos 3-5, 28029 Madrid, Spain; 3 Heart Failure and Transplantation Unit, Cardiology Department, University and Polytechnic La Fe Hospital, Avd. Fernando Abril Martorell 106, 46026 Valencia, Spain

**Keywords:** heart failure, sphingolipid metabolism, sphingosine-1-phosphate (S1P), ceramide synthase 1 (*CERS1*), ceramide/S1P rheostat

## Abstract

Disturbances in sphingolipid metabolism lead to biological function dysregulation in many diseases, but it has not been described in heart failure (HF). Sphingosine-1-phosphate (S1P) levels have not ever been measured in the myocardium. Therefore, we analyze the gene dysregulation of human cardiac tissue by mRNA-seq (*n* = 36) and ncRNA-seq (*n* = 50). We observed most major changes in the expression of genes belonging to de novo and salvage pathways, and the tight gene regulation by their miRNAs is largely dysregulated in HF. We verified using ELISA (*n* = 41) that ceramide and S1P accumulate in HF cardiac tissue, with an increase in the ceramide/S1P ratio of 57% in HF. Additionally, changes in left ventricular mass and diameters are directly related to *CERS1* expression and inversely related to S1P levels. Altogether, we define changes in the main components of the sphingolipid metabolism pathways in HF, mainly de novo and salvage, which lead to an increase in ceramide and S1P in cardiac tissue, as well as an increase in the ceramide/S1P ratio in HF patients. Therapeutic gene modulation focused on restoring ceramide levels or reversing the ceramide/S1P ratio could be a potential therapy to be explored for HF patients.

## 1. Introduction

Our understanding of cardiovascular diseases and their management has changed dramatically over the last 30 years with the identification of the various pathways that lead to their development and progression and the successful development of effective therapies that target them. This has led to a concomitant reduction in mortality and morbidity and an improvement in the functional capacity and quality of life of patients. However, there are still major unmet needs in the management of these diseases. They continue to be the number one cause of death globally, with their social and economic impact remaining largely unchanged. Therefore, there is a need to identify new therapeutic targets.

Sphingolipids are key signaling molecules that regulate a variety of biological functions and disturbances in sphingolipid metabolism that lead to aberrant apoptosis, autophagy, cell differentiation, and mitochondrial metabolism in many diseases, including cardiovascular diseases [1]. These defects induce alterations in cardiomyocyte structure and function [2], suggesting that sphingolipids likely contribute to cardiomyopathy through these mechanisms.

In recent years, the role of ceramide and sphingosine-1-phosphate (S1P) in the physiology and pathophysiology of the heart has attracted significant attention. The accumulation of specific ceramides in the ischemic myocardium that is further elevated upon subsequent reperfusion has been observed, indicating that ceramide may be involved in the induction of cardiomyocyte apoptosis by ischemia/reperfusion injury [3,4]. Similarly, the accumulation of lipids within the myocardium with non-ischemic heart failure (HF) occurs, and although the mechanism is unclear, it seems that the accumulation of ceramide plays a key role in this phenomenon [5,6] via the activation of programmed cell death [7] or its effect on myocardial glucose and fatty acid metabolism [6].

Meanwhile, evidence suggests that S1P acts as a cardioprotective agent that preserves the heart against ischemia/reperfusion injury [8]. However, lower S1P plasma concentrations have been observed in patients with acute myocardial infarction when compared with healthy controls (CNT), with a further reduction over the next few days [9], suggesting a sustained reduction in the protective effect of plasma S1P after infarction [10]. Nevertheless, we recently observed an increase in plasma S1P in patients experiencing acute rejection after heart transplantation, demonstrating a robust capability for detection that improves gradually with increasing severity [11].

Further studies are needed to define the specific lipid abnormalities that occur in human hearts at various stages of failure. Despite the relevance of sphingolipid metabolism, transcriptional changes in their key metabolic genes have not been reported for HF. Only once we understand the underlying molecular mechanism of sphingolipid metabolism and its key mediators can we develop a reasonable plan to prevent or treat HF. Given this, we designed this study to identify the alterations in sphingolipid metabolism at the mRNA level and their miRNA regulators in human cardiac tissue from patients with HF, as well as the levels of the main sphingolipids from the de novo, salvage, and sphingomyelin pathways in this tissue. We highlighted alterations in the de novo and the salvage pathways that lead to cardiac ceramide and S1P accumulation and to an increase in ceramide/S1P balance in HF patients, especially *CERS1*, whose expression is related to cardiac remodeling.

## 2. Materials and Methods

### 2.1. Source of Tissue Samples

Myocardial tissue samples were obtained from the left ventricle of 52 subjects: 42 patients with HF (non-ischemic dilated and ischemic cardiomyopathy patients) undergoing cardiac transplantation and 10 CNT samples from non-diseased donor hearts. All CNT donors had no history of heart disease with normal left ventricular (LV) function (>50%) determined by echocardiography. In all cases, the cause of death was a motor vehicle or cerebrovascular accident. The CNT hearts were considered unsuitable for cardiac transplantation donation because of blood type or size incompatibility.

Samples were taken from an area proximal to the left ventricle apex, were stored in 0.9% NaCl at 4 °C for a mean time of 4.4 ± 3.0 h after the loss of coronary circulation, and were then stored at −80 °C until RNA extraction. Our hospital has accomplished more than 700 transplants in the last 25 years and has been ranked as the national heart transplantation leader several times. In accordance, our samples are high quality with high RNA integrity number (RIN) values (greater or equal to 9). We have access to operating rooms during interventions and fully explanted hearts in all cases, so we standardize our methodology to choosing tissue samples from the same area of the left ventricle.

### 2.2. Patient Characteristics

Clinical history, electrocardiography, hemodynamic studies, Doppler echocardiography, and coronary angiography data were available. Table 1 shows the patient characteristics. Non-ischemic dilated cardiomyopathy was diagnosed when patients had intact coronary arteries ascertained by coronary angiography and LV systolic dysfunction (ejection fraction (LVEF) < 40%) with a dilated left ventricle (LV end-diastolic diameter (LVEDD) > 55 mm) assessed by echocardiography. Furthermore, none of the patients had reported a family history of the disease or showed evidence of significant primary valvular disease. Patients were diagnosed with ischemic cardiomyopathy based on the following inclusion criteria: (i) there were prior documented episodes of acute myocardial infarction, (ii) the echocardiography showed normal contractility segments coexisting with other dyskinetic or akinetic segments, and (iii) the electrocardiography showed signs of ischemia or myocardial necrosis. All patients have been classified according to the New York Heart Association (NYHA) functional criteria and were receiving medical treatment according to the guidelines of the European Society of Cardiology [12]. This study was approved by the Ethics Committee (Biomedical Investigation Ethics Committee of La Fe University Hospital of Valencia, Spain; Protocol Code 2016/0320, 15 November, 2016) and was conducted in accordance with the principles outlined in the Declaration of Helsinki [13], and all subjects gave written informed consent to participate in the study.

### 2.3. RNA Extraction and Integrity, mRNA-Seq, and ncRNA-Seq Analysis

RNA extraction, determination of purity and integrity of RNA samples, mRNA-seq, and ncRNA-seq analysis were performed as previously described by Tarazón et al. [14] and are extensively described in the Supplementary Materials.

### 2.4. Enzyme-Linked Immunosorbent Assay (ELISA)

Assessment of ceramide, sphingomyelin, sphingosine, and S1P levels in myocardial tissue homogenates was determined via specific sandwich enzyme-linked immunosorbent assay following the manufacturer’s instructions (Human Ceramide (CER) Elisa kit MBS7254089 from MyBioSource (San Diego, CA, USA) and Human Sphingomyelin (SPH) Elisa kit CEA805Ge, Human Sphingosine (Sph) Elisa kit CEB821Ge, and Human Sphingosine-1-Phosphate (S1P) Elisa kit CEG031Ge from Cloud-Clone Corp. (Houston, TX, USA)).

Briefly, twenty-five milligrams of frozen left ventricle was homogenized in an extraction buffer (2% SDS, 10 mM EDTA, 6 mM Tris–HCl, pH 7.4) in a FastPrep-24 homogenizer (MP Biomedicals, Santa Ana, CA, USA) with specifically designed Lysing Matrix D tubes. The homogenates were centrifuged, and the supernatant was aliquoted.

The ceramide test has a limit of detection up to 1.0 ng/mL, sphingomyelin up to 1.85 µg/mL, sphingosine up to 2.67 ng/mL, and S1P up to 4.62 ng/mL. The intra- and inter-assay coefficients of variation were 10% and 10–12%, respectively. No significant cross-reactivity or interference between these sphingolipids and analogs was observed. The tests were quantified at 450 nm in a dual-wavelength microplate reader (Sunrise; Tecan, Tecan Ibérica Instrumentación S.L Barcelona, Spain) using Magellan version 2.5 software (Tecan).

### 2.5. Statistical Methods

Data are presented as mean value ± standard deviation for continuous variables and as a percentage for discrete variables. The Kolmogorov–Smirnov test was used to analyze the distribution of the variables. Comparisons of clinical characteristics were achieved using Student’s *t*-test for continuous variables and Fisher’s exact test for discrete variables. Comparisons of mRNAs, miRNAs, and sphingolipids tissue levels were performed using the Mann–Whitney U test. Finally, Pearson’s correlation coefficients were calculated to determine the relationships among levels of molecules and clinical characteristics. Significance was assumed as *p* < 0.05. All statistical analyses were performed using SPSS software v. 20 for Windows (version 20.0; IBM SPSS Inc.; Endicott, NY, USA).

## 3. Results

### 3.1. Clinical Characteristics of HF Patients

The study populations for each assay are described in Table 1. They are homogeneous populations based on the clinical characteristics of the patients. We analyzed a total of 52 myocardial human heart samples from 42 patients undergoing transplantation after being diagnosed with non-ischemic DCM or ICM cardiomyopathy, while the CNT samples were obtained from 10 non-diseased donor hearts.

Most of the patients were men (93%), and their mean age was 53 ± 10 years. The patients all presented with an NYHA functional classification between III and IV and had previously been diagnosed with significant comorbidities, including hypertension (31%), hypercholesterolemia (17%), and diabetes mellitus (30%). The CNT group mainly comprised men (62%) and had a mean age of 49 ± 15 years. Comorbidities and other echocardiographic data were not available for the CNT group, in accordance with the Spanish Organic Law on Data Protection 15/1999.

### 3.2. mRNA Expression of the Sphingolipid Metabolism Genes

**Figure 1:**
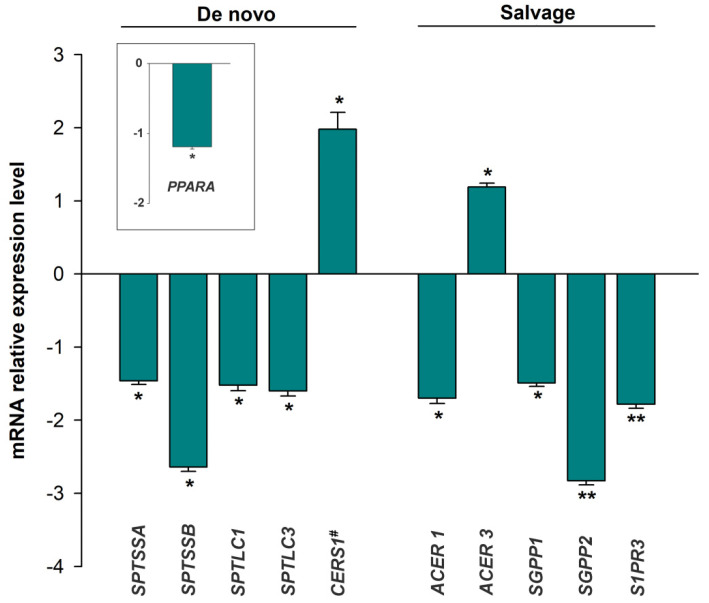
Differential expression of genes involved in sphingolipid metabolism grouped according to their metabolic pathways that converge upon ceramide: de novo and salvage pathways. The values from the controls (*n* = 10) were set to 1. Data are presented as the fold change ± standard error. Heart failure patients (*n* = 26; green bars). Mann–Whitney U test: * *p* < 0.05, ** *p* < 0.01 vs. control group. # This enzyme acts both in the de novo biosynthesis and in the salvage pathway (Table S1). PPARA is a key regulator of lipid metabolism through the de novo ceramide pathway (transcription factor).

We performed a transcriptomic analysis using mRNA-seq on cardiac tissue (HF, *n* = 26; CNT, *n* = 10) to identify differentially expressed genes associated with HF. When we focused on the genes involved in sphingolipid metabolism (Table S1), we found 12 differentially expressed genes in HF patients compared to CNTs (*p* < 0.05) (Figure S1). When focusing on each of the metabolic pathways that converge upon ceramide, we found differences in the expression of genes that participate mainly in the de novo and salvage pathways (Figure 1).

In the de novo biosynthesis of sphingolipids, we found that the serine palmitoyl transferases that catalyze the first step of this process, the condensation of serine and palmitoyl CoA to produce 3-ketodihydrosfingosine, were downregulated (*SPTSSA*: −1.46 ± 0.25, *p* = 0.022; *SPTSSB*: −2.46 ± 0.21, *p* = 0.035; *SPTLC1:* −1.52 ± 0.37, *p* = 0.049; *SPTLC3:* −1.60 ± 0.36, *p* = 0.031) and that transcription factor peroxisome proliferator-activated receptor-alpha, which is a key regulator of lipid metabolism, was also downregulated (*PPARA*: −1.19 ± 0.17, *p* = 0.011) in HF tissues. The levels of this transcription factor were also different between patients diagnosed with hypercholesterolemia and those without (1.19 ± 0.03, *p* = 0.040) and were shown to correlate with blood cholesterol levels (*r* = −0.449, *p* = 0.036) (Figure S2). However, ceramide synthase 1, which acts in both the de novo biosynthesis and the salvage pathway, catalyzing the formation of ceramide from sphinganine or sphingosine, was increased in patients with HF (*CERS1*: 1.98 ± 1.05, *p* = 0.040) (Figure 1).

In the salvage pathway, we found that ceramidase, alkaline ceramidase 1 (*ACER1*, −1.70 ± 0.28; *p* = 0.047), which catalyzes the hydrolysis of ceramide to sphingosine, was downregulated in HF tissue, while alkaline ceramidase 3 (*ACER3*: 1.19 ± 0.26, *p* = 0.029) was upregulated in these tissues. Furthermore, the enzymes that dephosphorylate S1P, sphingosine-1-phosphate phosphatase 1 and 2 (*SGPP1*: −1.49 ± 0.24, *p* = 0.044; *SGPP2:* −2.83 ± 0.28, *p* = 0.002), and S1P receptor 3 (*S1PR3*: −1.78 ± 0.28, *p* = 0.003) were all downregulated in HF patients (Figure 1).

The synthesis of sphingomyelin from ceramide, via the sphingomyelinase or the hydrolysis pathway, was also shown to be impacted by HF, with a marked decrease in the expression of the gene encoding sphingomyelin synthase 1 (*SGMS1*: −1.66 ± 0.25, *p* = 0.006). We did not find any differentially expressed mRNAs for the regulatory genes controlling sphingolipid metabolism.

### 3.3. Expression of the miRNAs Involved in the Regulation of Sphingolipid Metabolism

**Figure 2:**
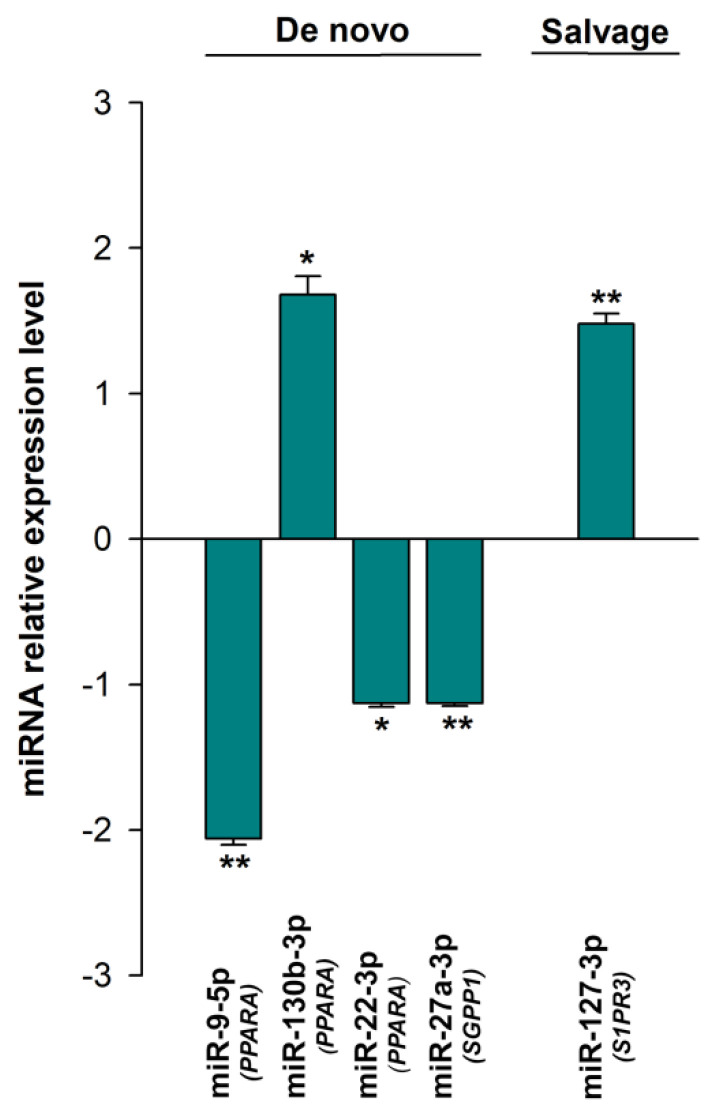
Differential expression of the miRNAs involved in the post-transcriptional regulation of sphingolipid metabolism grouped according to the metabolic pathways of their targets: de novo and salvage pathways. The values from the controls (*n* = 8) were set to 1. Data are presented as the fold change ± standard error. Heart failure patients (HF, *n* = 42; green bars). Mann–Whitney U test: * *p* < 0.05, ** *p* < 0.01 vs. the control group. In parentheses are the target genes for each miRNA.

We then went on to use non-coding RNA sequencing (ncRNA-seq) to identify the differentially expressed miRNAs involved in the post-transcriptional regulation of the sphingolipid metabolism genes (HF, *n* = 42; CNT, *n* = 8). To do this, we went on to evaluate the expression of previously identified miRNAs linked to sphingolipid metabolism in our data set (Table S2). As we show in the table, we observed that many of the genes involved in the metabolism of ceramides are subject to close regulation by various miRNAs in both the de novo and salvage pathways and other regulators involved in the synthesis of ceramide. Thus, we found that HF is strongly affected by miRNA dysregulation during sphingolipid metabolism, with alterations in several miRNAs linked to the deregulated mRNA of the de novo (miR-9-5p, miR-130b-3p, miR-22-3p, and miR-27a-3p) and salvage pathways (miR-27a-3p) (Figure 2). There were also a handful of miRNAs associated with other regulatory genes (miR-127-3p, miR-490-3p). We did not find any differentially expressed miRNAs targeting the hydrolysis pathway of sphingolipid metabolism.

### 3.4. Sphingolipid Levels in Heart Tissue

**Figure 3:**
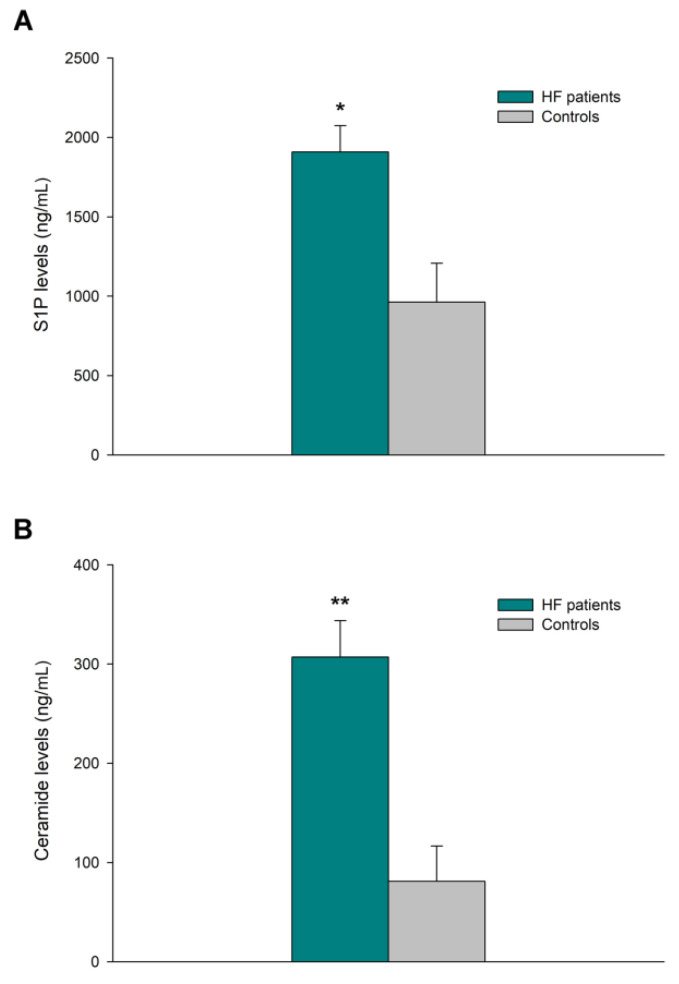
Differential expression of bioactive sphingolipids in cardiac tissue. (**A**) Sphingosine-1-phosphate (S1P) and (**B**) ceramide levels in heart failure (HF) and healthy controls. Data are presented as the mean ± standard error. HF patients (*n* = 36; green bars) and controls subjects (*n* = 5; gray bars). Mann–Whitney U test: * *p* < 0.05, ** *p* < 0.01 vs. the control group.

The concentration of bioactive sphingolipids S1P, ceramide, sphingosine, and sphingomyelin was determined using ELISA (HF, *n* = 36; CNT, *n* = 5). S1P and ceramide were shown to be dysregulated in HF patients (1908 ± 625 vs. 963 ± 489 ng/mL of homogenate, *p* = 0.014, Figure 3A, and 307 ± 218 vs. 81 ± 79 ng/mL of homogenate, *p* = 0.004, Figure 3B, respectively), and the tissue ceramide/S1P ratio was increased by 57% in this group of samples.

### 3.5. Relationships between Molecular Heart Tissue Levels and Ventricular Parameters of HF Patients

We investigated the potential relationships between mRNA expressions of sphingolipid metabolism genes, sphingolipid concentrations, and ventricular parameters of HF patients (Table 2). Interestingly, differential mRNA expression of ceramide synthase 1 was associated with changes in LV mass (r = 0.797, *p* < 0.0001) and LV end-systolic (LVESD, r = 0.561 and *p* = 0.012) and end-diastolic diameters (LVEDD, r = 0.601 and *p* = 0.007). S1P levels were shown to inversely correlated with these parameters (LVESD, r = −0.552 and *p* = 0.041; LVEDD, r = −0.541 and *p* = 0.046), and a reverse trend was observed for LV mass (r = −0.550, *p* = 0.052). Both *CERS1* and S1P also demonstrated a good relationship (r = −0.797 and *p* = 0.006). S1P levels also showed a remarkable relationship with the ceramide levels in this tissue (r = 0.915 and *p* < 0.0001).

## 4. Discussion

Sphingolipid metabolism in the heart is of considerable interest because of its involvement in cardiac pathology. However, most studies have examined the role of individual bioactive sphingolipids in the pathophysiology of various heart diseases without examining the metabolic pathways that lead to these abnormalities. Therefore, they remain largely undefined [10]. Although many of the molecules involved in sphingolipid metabolism have well-understood physiological and pathophysiological mechanisms, their status in human HF has not been described. In fact, S1P levels in HF tissue have never been reported in the literature.

This study defined the expression of various genes involved in the metabolism of the sphingolipids, revealing the alteration of key components, mainly in the de novo and salvage pathways, which lead to the accumulation of ceramide and S1P in the cardiac tissue of patients with advanced-stage chronic HF. These changes help to maintain the increased ceramide-S1P balance in these patients. S1P accumulation is also associated with cardiac remodeling in HF patients.

Intrinsic cardiac metabolism in the adult heart depends primarily on the utilization of fatty acids for oxidative phosphorylation and ATP production, but during stress, the heart prefers to switch to glucose for energy generation. As a result, lipids accumulate in the failing myocardium [15]. Given this, our results demonstrate an accumulation of ceramide in patients with HF, as has been described in several models of cardiac lipotoxicity [16]. This lipid regulates, through many key intracellular effectors, the apoptotic program [17]. Conversely, numerous studies have revealed that S1P, which participates in cardiovascular function through many different processes, possesses cardioprotective properties, mainly protecting cardiomyocytes against apoptosis [18]. Thus, alterations in the plasma levels of S1P have been observed in several cardiovascular disorders [19]. However, to the best of our knowledge, this is the first study to analyze the levels of S1P in cardiac tissue. We observed higher levels of cardiac S1P in HF patients, which could be part of a compensatory effect designed to combat the increase in ceramide levels and its apoptotic effect [20]. This premise is supported by the relationship between S1P and cardiac remodeling, where higher levels of S1P were associated with smaller LV diameters and reduced LV mass. In fact, it has been suggested that the dynamic balance between the intracellular levels of ceramide and S1P determines cell fate [20]. Although we observed an increase in both lipids in HF patients, the ceramide/S1P ratio increased in these patients, suggesting that the accumulation of S1P may not be sufficient to counteract the effects of ceramide-mediated apoptosis [21].

The intrinsic metabolism of each tissue type helps to determine the levels of metabolites present. However, changes in these levels can also be the result of variation in the expression of the enzymes involved in response to organ damage [22]. Thus, in response to a variety of stresses such as serum deprivation [23], oxidative stress [24], or photodamage [25,26], sphingomyelin synthase inhibition, ceramide accumulation, and induced apoptosis occur. In this sense, we observed a reduction in sphingomyelin synthase, *SGMS1,* but not *SGMS2*, in patients with HF. Li et al. already observed that siRNA treatment targeting *SGMS1* significantly increased cellular ceramide contents in cultured cells, while siRNA targeting *SGMS2* did not result in the same outcome [27]. In turn, siRNA-treated HeLa cells showed a similar increase in ceramide levels (*SGMS1* and *SGMS2* siRNAs), but *SGMS1* represents the major enzymatic activity, being the main Golgi-associated sphingomyelin synthases, while *SGMS2* is the principal plasma membrane-associated sphingomyelin synthase [28].

On the other hand, peroxisome proliferator-activated receptor is involved in the control of several pathways of lipid synthesis or catabolism via the regulation of the gene expression level of several key lipid-metabolizing enzymes. Specifically, *PPARA* participates in the regulation of the first step in the de novo biosynthesis of sphingolipid by modulating serine palmitoyltransferase [29]. We observed a reduction in the expression of the transcription factor *PPARA,* along with a reduction in the majority of the serine palmitoyltransferase enzyme complex (*SPTSSA*, *SPTSSB*, *SPTLC1*, and *SPTLC3*). Patients with HF present with a reduction in PPARα protein levels, suggesting the important role of this transcription factor in the reduction of fatty acid utilization in adult heart tissue during cardiac injury [30]. In this sense, we observed an increase in plasma cholesterol levels which are linked to the reduced expression of *PPARA*, and we observed significant differences in its expression in patients with hypercholesterolemia compared to those patients who do not present this comorbidity. Further, it has been observed that functional inhibition of the serine palmitoyltransferase enzyme complex in the animal model of ischemic LV dysfunction increases expression of ceramide synthase 1 and is accompanied by an increase in cardiac-specific ceramide levels [31]. Our study showed a reduction in the expression of this enzyme complex, which was accompanied by an increase in ceramide levels and the expression of *CERS1*. Moreover, we found that the expression levels of *CERS1* were related to cardiac remodeling, with higher levels of expression in patients with increased ventricular diameters and mass.

We previously observed that alterations in the salvage pathway could lead to the accumulation of S1P. Specifically, we found a reduction in the expression of *SGPP1* and *SGPP2* without changes in the expression of *SPHK*s or *SGPL1* [11]. Thus, here we confirm that the alterations found in these enzymes lead to an increase in the production of S1P that accumulates in the myocardial tissue and is also linked to the increase in the ceramide content of this tissue. Likewise, we think that the enzymes involved in the ceramide/sphingosine balance, mainly the alterations found in the levels of *ACER1*, *ACER3*, and *CERS1*, contribute to the increased levels of ceramide and S1P that we found in HF.

Although there is little information describing the miRNAs regulating sphingolipid metabolism, several miRNAs that regulate genes involved in both the de novo and salvage pathways and their regulation have been described. Understanding the mechanisms regulating sphingolipid biosynthesis in HF may provide novel information that might be useful when developing therapeutic interventions. In addition to the alteration of key components of these pathways, our data also indicate that HF is affected by miRNA dysregulation, which could also contribute to the accumulation of ceramide and S1P, and most importantly to the rheostat ceramide/S1P associated with this syndrome.

A common limitation of studies that examine cardiac tissues from end-stage human HF is the extensive variability between individuals and their treatment, some of which might influence the results. Thus, all individuals evaluated here were receiving medical treatment according to the guidelines of the European Society of Cardiology [12]. Furthermore, tissue samples were taken from the transmural left ventricle apex; therefore, our findings cannot be generalized to all layers and regions of the left ventricle.

## 5. Conclusions

In this study, we outline the alterations in the key components of sphingolipid metabolism, mainly in the de novo and the salvage pathways, which leads to cardiac ceramide and S1P accumulation, as well as an increase in the ceramide/S1P ratio in HF patients. Therapeutic gene modulation focused on restoring ceramide levels or reversing the ceramide/S1P ratio could be a potential therapy to explore for HF. Particularly, *CERS1* could be an excellent candidate to explore for the treatment of HF patients due to its relationship with the dimensions of the left ventricle.

## Figures and Tables

**Table 1:** Clinical characteristics of heart failure patients.

	mRNA-Seq (*n* = 26)	ncRNA-Seq (*n* = 42)	ELISA (*n* = 36)
Age (years)	53 ± 9	52 ± 10	52 ± 10
Gender male (%)	96	93	87
NYHA class	III-IV	III-IV	III-IV
BMI (kg/m^2^)	27 ± 5	26 ± 4	27 ± 6
Hemoglobin (mg/dL)	14 ± 3	13 ± 2	14 ± 2
Hematocrit (%)	40 ± 7	40 ± 6	41 ± 5
Total cholesterol (mg/dL)	155 ± 39	159 ± 45	164 ± 50
Prior hypertension (%)	25	31	21
Prior smoking (%)	71	71	75
Diabetes mellitus (%)	29	30	35
LVEF (%)	21 ± 8	21 ± 8	22 ± 9
LVESD (mm)	66 ± 12	61 ± 12	63 ± 12
LVEDD (mm)	74 ± 11	69 ± 12	71 ± 11
Left ventricular mass (g)	362 ± 142	316 ± 120	341 ± 109
Left ventricle mass index (g/m^2^)	194 ± 76	166 ± 60	180 ± 65
Duration of disease (months) ^#^	59 ± 56	44 ± 38	57 ± 52

NYHA, New York Heart Association; BMI, body mass index; LVEF, left ventricular ejection fraction; LVESD, left ventricular end-systolic diameter; LVEDD, left ventricular end-diastolic diameter. ^#^ Duration of disease from diagnosis of heart failure until heart transplant.

**Table 2:** Relationships between mRNA expressions of sphingolipid metabolism genes, sphingolipid levels, and ventricular parameters in heart failure patients.

	LV Mass	LVESD	LVEDD
*CERS1*	r = 0.797 *p* < 0.0001	r = 0.561 *p* = 0.012	r = 0.601 *p* = 0.007
S1P	r = −0.550 *p* = 0.052	r = −0.552 *p* = 0.041	r = −0.541 *p* = 0.046

LV mass, left ventricular mass; LVESD, left ventricular end-systolic diameter; LVEDD, left ventricular end-diastolic diameter.

## Data Availability

The mRNA-seq data discussed in this publication have been deposited in NCBI’s Gene Expression Omnibus [32] and are accessible through GEO Series Accession Number GSE55296 (http://www.ncbi.nlm.nih.gov/geo/query/acc.cgi?acc=GSE55296, accessed on 28 April 2014).

## Supplementary Materials

The following are available online at https://www.mdpi.com/article/10.3390/biomedicines10010135/s1, RNA extraction and integrity, mRNA-seq, and ncRNA-seq analysis methods. Figure S1. String interaction network (https://string-db.org/; accessed on 13 October 2021). of 12 genes deregulated in heart failure patients Figure S2. Differential expression levels of *PPARA* between heart failure patients with hypercholesterolemia and those without (A). Correlation between *PPARA* and cholesterol levels in patients with heart failure, (B). Table S1. mRNA expression of sphingolipid metabolism genes in heart failure. Table S2. miRNA involved in the regulation of the expression of sphingolipid metabolism genes.
