# Supplementary material for: Implication of Sphingolipid Metabolism Gene Dysregulation and Cardiac Sphingosine-1-Phosphate Accumulation in Heart Failure
Source: Biomedicines. 2022 Jan 8;10(1):135. doi: 10.3390/biomedicines10010135 (PMC8773611; doi:10.3390/biomedicines10010135)
Supplement: Supplementary file 1 [file biomedicines-10-00135-s001.zip › biomedicines-1513117-supplementary.pdf]

Supplementary Materials

Implication of sphingolipid metabolism gene dysregulation and cardiac sphingosine-1-phosphate accumulation in heart failure

Lorena Pérez-Carrillo[1], PhD; Isaac Giménez-Escamilla[1], PhD; Luis Martínez-Dolz[1,2], MD; Ignacio José Sánchez-Lázaro[1,2], MD, Manuel Portolés[1], PhD; Esther Roselló-Lletí[1†*], PhD; Estefanía Tarazón[1†*], PhD.

[1]Myocardial Dysfunction and Cardiac Transplantation Unit, Health Research Institute Hospital La Fe (IIS La Fe, Valencia, Spain; and CIBERCV, Madrid, Spain. [2]Heart Failure and Transplantation Unit, Cardiology Department, University and Polytechnic La Fe Hospital, Valencia, Spain. [†]These authors jointly supervised this work.

MATERIALS AND METHODS

RNA extraction and integrity

Heart samples were homogenized in a TissueLysser LT (Qiagen). RNA extractions were performed using a PureLink™ Kit (Ambion Life Technologies) for mRNA sequencing (mRNA-seq) or the Quik-RNA™ miniprep plus kit (Zymo Research) for non-coding RNA sequencing (ncRNA-seq), according to the manufacturer's instructions. RNA was quantified using a NanoDrop1000 spectrophotometer and at Qubit 3.0 fluorometer (Thermo Fisher Scientific). The purity and integrity of RNA samples was determined using an Agilent 2100 Bioanalyzer with the RNA 6000 Nano LabChip kit (Agilent Technologies). All samples displayed a 260/280 absorbance ratio > 2.0 and RNA integrity numbers ≥9.

mRNA-seq analysis

PolyA-RNA was isolated form 25 micrograms of total RNA using the MicroPoly(A) Purist kit (Ambion). Total Poly(A) RNA samples were used to generate whole transcriptome libraries for sequencing on the SOLiD 5500XL platform, following the manufacturer's recommendation (Life Technologies). No RNA spike-in controls was used. Amplified cDNA quality was analyzed by the Bioanalyzer 2100 DNA 1000 kit (Agilent Technologies) and quantified using the Qubit 2.0 Fluorometer (Invitrogen). The whole transcriptome libraries were used for making SOLiD templated beads following the SOLiD Templated Bead Preparation guide. Bead quality was estimated based on WFA (workflow analysis) parameters. The samples were sequenced using the 50625 paired-end protocol, generating 75 nt +35 nt (Paired-End) +5 nt (Barcode) sequences. Quality data were measured using software SETS parameters (SOLiD Experimental Tracking System).

The initial whole transcriptome paired-end reads obtained from sequencing were mapped against the latest version of the human genome (version GRchr37/hg19) using the Life Technologies mapping algorithm (http://www.lifetechnologies.com/), version 1.3. It was using

the standard Bioscope parameters of version 1.3, in paired ends and whole transcriptome analysis. For both reads, forwards and revers, the seed was the first 25 nucleotides with a maximum of 2 mismatches allows. The aligned records were reported in BAM/SAM format [1]. Bad quality reads (Phred score <10) were eliminated using Picard Tools software, version 1.83 (http://broadinstitute.github.io/picard/).

Subsequently, gene prediction were estimated using the cufflinks method [2] and the expression levels were calculated using the HTSeq software, version 0.5.4p3 [3], this method eliminate the multimapped reads, only the unique reads are considered for gene expression estimation. The edgeR method, version 3.2.4, was applied for differential expression analysis between conditions [4]. This method rely on different normalize process based in depth global samples, CG composition and length of genes. In the differential expression process, this method relies on a Poisson model to estimate the variance of the RNA-seq data for differential expressions. The mRNA-seq data discussed in this publication have been deposited in NCBI's Gene Expression Omnibus [5] and are accessible through GEO Series accession number GSE55296 (http://www.ncbi.nlm.nih.gov/geo/query/acc.cgi?acc=GSE55296).

ncRNA-seq analysis

The cDNA libraries have been obtained following Illumina´s recommendations. Briefly, 3´ and 5´adaptors were sequentially ligated to the RNA prior to reverse transcription and cDNA generation. The cDNA was enriched using PCR to create an indexed double-stranded cDNA library, and size selection was performed using a 6% polyacrylamide gel. The quality and quantity of the libraries were analysed using a 4200 TapeStation D1000 High-Sensitivity assay. The cDNA libraries were pooled and the pools were sequenced using paired-end sequencing (100 x 2) in the Illumina HiSeq 2500 sequencer.

The quality control of the raw data was performed using the FastQC tool. For the adapter and quality fillert of raw data trim_galore was applied [http://www.bioinformatics.babraham.ac.uk/projects/trim_galore/]. Then, the insufficient

quality reads (phred score < 20) were eliminated using the Picard Tools software [6]. RNAs predictions were estimated using HT Seq software (version 0.6.0) [3]. Differential expression analysis between conditions was assessed using DESeq2 method [7] (version 3.4). We considered as differently expressed ncRNAs those with a P value (P adj) corrected by FDR ≤ 0.05 to avoid identification of false positives across the differential expression data [8].

FIGURES

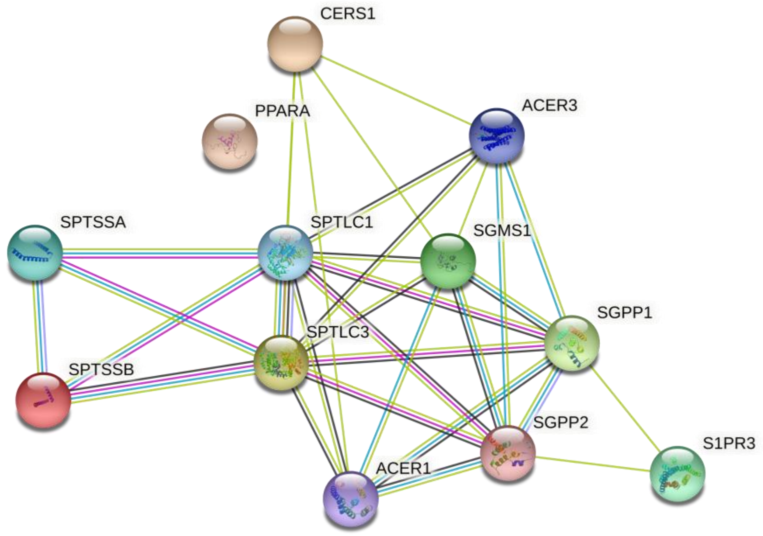

Figure S1. String interaction network (https://string-db.org/) of 12 genes deregulated in heart failure patients. Only interactions with > 0.4 confidence interaction scores are shown. Know protein interaction (experimentally demonstrated) (*pink*) and from curated databases (*turquoise*), gene co- expression (*black*), textmining (cited together in PubMed abstracts) (*green*), protein homology (*light purple*). Proteins with known structures are indicated.

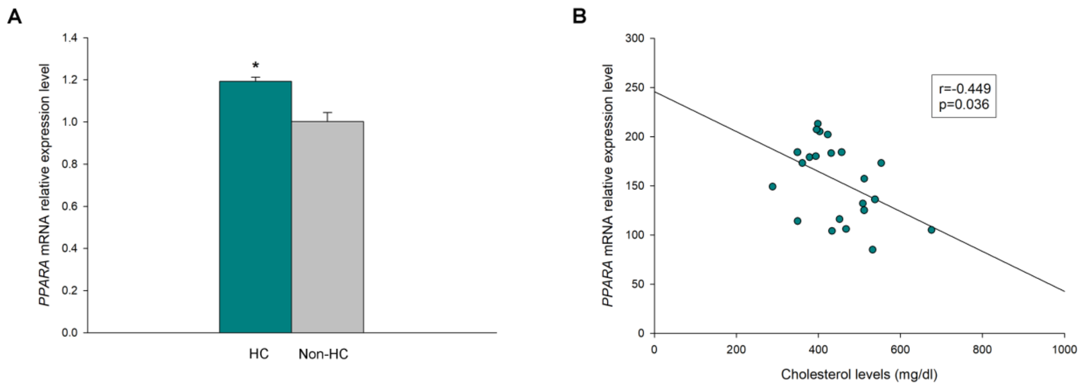

Figure S2. Differential expression levels of *PPARA* between heart failure patients with hypercholesterolemia and those without (A). Correlation between *PPARA* and cholesterol levels in patients with heart failure, (B). The values from the non-hypercholesterolemia patients were set to 1. Data are presented as the fold change ± standard error. Heart failure hypercholesterolemia patients (HC, n=3; green bars) and heart failure non-hypercholesterolemia patients (Non-HC, n=21; grey bars). Mann-Whitney U test and Pearson correlation: *p<0.05.

TABLES

Table S1. mRNA expression of sphingolipid metabolism genes in heart failure.

Ensembl	Gene	Description
		**De novo pathway**
**ENSG00000165389**	***SPTSSA***	**Serine palmitoyltransferase small subunit A**
**ENSG00000196542**	***SPTSSB***	**Serine palmitoyltransferase small subunit B**
**ENSG00000090054**	***SPTLC1***	**Serine palmitoyltransferase 1**
ENSG00000100596	*SPTLC2*	Serine palmitoyltransferase 2
**ENSG00000172296**	***SPTCL3***	**Serine palmitoyltransferase 3**
ENSG00000119537	*KDSR*	3-ketodihydrosphingosine reductase
**ENSG00000223802**	***CERS1***	**Ceramide synthase 1***
ENSG00000143418	*CERS2*	Ceramide synthase 2*
ENSG00000154227	*CERS3*	Ceramide synthase 3*
ENSG00000090661	*CERS4*	Ceramide synthase 4*
ENSG00000139624	*CERS5*	Ceramide synthase 5*
ENSG00000172292	*CERS6*	Ceramide synthase 6*
ENSG00000149926	*TLCD3B*	Ceramide synthase*
ENSG00000143753	*DEGS1*	Sphingolipid delta(4)-desaturase DES1
ENSG00000168350	*DEGS2*	Sphingolipid delta(4)-desaturase/C4-monooxygenase DES2
**ENSG00000186951**	***PPARA***	**Peroxisome proliferator-activated receptor alpha (PPAR-alpha)**
ENSG00000112033	*PPARD*	Peroxisome proliferator-activated receptor delta (PPAR-delta)
ENSG00000132170	*PPARG*	Peroxisome proliferator-activated receptor gamma (PPAR-gamma)

	**Salvage pathway**	
**ENSG00000167769**	***ACER1***	**Alkaline ceramidase 1 (AlkCDase 1)**
ENSG00000177076	*ACER2*	Alkaline ceramidase 2 (AlkCDase 2)
**ENSG00000078124**	***ACER3***	**Alkaline ceramidase 3 (AlkCDase 3)**
ENSG00000104763	*ASAH1*	Acid ceramidase (ACDase)
ENSG00000188611	*ASAH2*	Neutral ceramidase (NCDase)
ENSG00000177076	*SPHK1*	Sphingosine kinase 1
ENSG00000063176	*SPHK2*	Sphingosine kinase 2
ENSG00000153820	*SPHKAP*	A-kinase anchor protein SPHKAP
**ENSG00000126821**	***SGPP1***	**Sphingosine-1-phosphate phosphatase 1**
**ENSG00000163082**	***SGPP2***	**Sphingosine-1-phosphate phosphatase 2**
ENSG00000166224	*SGPL1*	Sphingosine-1-phosphate lyase 1
ENSG00000170989	*S1PR1*	Sphingosine 1-phosphate receptor 1
ENSG00000267534	*S1PR2*	Sphingosine 1-phosphate receptor 2
**ENSG00000213694**	***S1PR3***	**Sphingosine 1-phosphate receptor 3**
ENSG00000125910	*S1PR4*	Sphingosine 1-phosphate receptor 4
ENSG00000180739	*S1PR5*	Sphingosine 1-phosphate receptor 5
ENSG00000148154	*UGCG*	Ceramide glucosyltransferase
ENSG00000177628	*GBA*	Lysosomal acid glucosylceramidase
ENSG00000070610	*GBA2*	Non-lysosomal glucosylceramidase
	**Sphingomyelinase or hydrolysis pathway**	
ENSG00000166311	*SMPD1*	Sphingomyelin phosphodiesterase (aSMase)
ENSG00000135587	*SMPD2*	Sphingomyelin phosphodiesterase 2 (nSMase)
ENSG00000103056	*SMPD3*	Sphingomyelin phosphodiesterase 3 (nSMase2)
ENSG00000136699	*SMPD4*	Sphingomyelin phosphodiesterase 4 (nSMase3)

**ENSG00000198964**	***SGMS1***	**Phosphatidylcholine:ceramide cholinephosphotransferase 1 (Sphingomyelin synthase 1)**
ENSG00000164023	*SGMS2*	Phosphatidylcholine:ceramide cholinephosphotransferase 2 (Sphingomyelin synthase 2)
	**Others**	
ENSG00000100422	*CERK*	Ceramide kinase (hCERK)
ENSG00000067113	*PLPP1*	Phospholipid phosphatase 1
ENSG00000141934	*PLPP2*	Phospholipid phosphatase 2
ENSG00000162407	*PLPP3*	Phospholipid phosphatase 3
ENSG00000224051	*CPTP*	Ceramide-1-phosphate transfer protein
ENSG00000113163	*CERT1*	Ceramide transfer protein (hCERT)
ENSG00000116478	*HDAC1*	Histone deacetylase 1 (HD1)
ENSG00000196591	*HDAC2*	H istone deacetylase 2 (HD2)

*These enzymes act both in the de novo biosynthesis and in the salvage pathway catalyzing formation of ceramide from sphinganine or sphingosine. The genes altered in the mRNA-seq study are highlighted in bold.

Table S2. miRNA involved in the regulation of the expression of sphingolipid metabolism genes.

Ensembl	miRNA	Target gene	References
	**De novo pathway**		
ENSG00000276365	miR-145	***SPTLC1***	[9]
ENSG00000284202	miR-137-3p		[10]
ENSG00000207613	miR-181c		
ENSG00000207598	miR-124-3p#	***SPTLC2***	[11]
**ENSG00000207933**	**miR-9#**		[10]
ENSG00000284032	miR-29a#		
ENSG00000283797	miR-29b-1		
ENSG00000283863	miR-3960	***CERS1***	[12]
ENSG00000284351	miR-3622a-5p	***CERS2***	[13]
**ENSG00000207933**	**miR-9-5p#**		[14]
**ENSG00000207870**	**miR-221-3p**		[15]
ENSG00000207725	miR-222-3p		
ENSG00000284190	miR-21-5p	***PPARA***	[16]
**ENSG00000207933**	**miR-9-5p#**		[17]
**ENSG00000283871**	**miR-130b-3p#**		[18]
ENSG00000199085	miR-148a-3p		[19]
ENSG00000284010	miR-675-5p		[20]
ENSG00000207708	miR-141-3p		[21]
**ENSG00000283824**	**miR-22-3p**		[22]
ENSG00000284032	miR-29a-3p#	***PPARD***	[23]
**ENSG00000207808**	**miR-27a-3p#**	***PPARG***	[24]
ENSG00000207654	miR-128-3p		[25]

ENSG00000208009	miR-130a-3p		[26]
**ENSG00000283871**	**miR-130b-3p#**		
**Salvage pathway**			
ENSG00000283733	miR-146a-5p	***ACER3***	[27]
ENSG00000266643	miR-3677-3p	***SPHK1***	[28]
**ENSG00000284544**	**miR-330-3p**		[29]
**ENSG00000207651**	**miR-28-5p**		[30]
ENSG00000207731	miR-506-3p		[31]
ENSG00000284321	miR-124-3p#		[32]
ENSG00000207983	miR-613		[33]
ENSG00000207696	miR-659-3p		[34]
**ENSG00000283604**	**miR-338-3p**	***SPHK2***	[35]
**ENSG00000207808**	**miR-27a-3p#**	***SGPP1***	[36]
ENSG00000207807	miR-95-3p		[37]
ENSG00000199177	miR-31-5p	***SGPP2***	[38]
ENSG00000207971	miR-125b-5p	***SGPL1***	[39]
ENSG00000284499	miR-363-3p	***S1PR1***	[40]
**ENSG00000207726**	**miR-455-5p**		[41]
ENSG00000207975	miR-181b-5p		[42]
**ENSG00000283904**	**miR-155-5p**		[43]
**ENSG00000199080**	**miR-133b**		[44]
**ENSG00000284204**	**miR-19a-3p**		[45]
ENSG00000207784	miR-542-3p		[46]
**ENSG00000207971**	**miR-125b-1-3p**		[47]
ENSG00000199161	miR-126a-3p	***S1PR2***	[48]
ENSG00000207611	miR-149-5p		[49]

ENSG00000207743	miR-495-3p	*S1PR3*	[50]
**ENSG00000207608**	**miR-127-3p#**		[51]
ENSG00000284565	miR-451a		[52]
	**Others**		
ENSG00000284357	miR-34a-5p#	*CERK*	[53]
**ENSG00000207608**	**miR-127-3p#**		[54]
ENSG00000284357	miR-34a-5p#	*HDAC1*	[55]
ENSG00000207714	miR-584-5p		[56]
ENSG00000198983	miR-449a		[57]
ENSG00000207735	miR-520d-5p		[58]
ENSG00000207785	miR-500a-5p	*HDAC2*	[59]
**ENSG00000207597**	**miR-490-3p**		[60]
ENSG00000283705	miR-92a-3p		[61]

#These miRNAs have several mRNA target of sphingolipid metabolism as reflected in the table.

The miRNA altered in the ncRNA-seq study are highlighted in bold.

REFERENCES

1. Li, H.; Handsaker, B.; Wysoker, A.; Fennell, T.; Ruan, J.; Homer, N.; Marth, G.; Abecasis, G.; Durbin, R.; Genome Project Data Processing, S. The Sequence Alignment/Map format and SAMtools. *Bioinformatics* **2009**, *25*, 2078-2079, doi:10.1093/bioinformatics/btp352.
2. Trapnell, C.; Williams, B.A.; Pertea, G.; Mortazavi, A.; Kwan, G.; van Baren, M.J.; Salzberg, S.L.; Wold, B.J.; Pachter, L. Transcript assembly and quantification by RNA-Seq reveals unannotated transcripts and isoform switching during cell differentiation. *Nature biotechnology* **2010**, *28*, 511-515, doi:10.1038/nbt.1621.
3. Anders, S.; Pyl, P.T.; Huber, W. HTSeq--a Python framework to work with high-throughput sequencing data. *Bioinformatics* **2015**, *31*, 166-169, doi:10.1093/bioinformatics/btu638.
4. Robinson, M.D.; McCarthy, D.J.; Smyth, G.K. edgeR: a Bioconductor package for differential expression analysis of digital gene expression data. *Bioinformatics* **2010**, *26*, 139-140, doi:10.1093/bioinformatics/btp616.
5. Edgar, R.; Domrachev, M.; Lash, A.E. Gene Expression Omnibus: NCBI gene expression and hybridization array data repository. *Nucleic acids research* **2002**, *30*, 207-210, doi:10.1093/nar/30.1.207.
6. Langmead, B.; Trapnell, C.; Pop, M.; Salzberg, S.L. Ultrafast and memory-efficient alignment of short DNA sequences to the human genome. *Genome biology* **2009**, *10*, R25, doi:10.1186/gb-2009-10-3-r25.
7. Love, M.I.; Huber, W.; Anders, S. Moderated estimation of fold change and dispersion for RNA-seq data with DESeq2. *Genome biology* **2014**, *15*, 550, doi:10.1186/s13059-014-0550-8.
8. Benjamini, Y.; Hochberg, Y. Controlling the False Discovery Rate: A Practical and Powerful Approach to Multiple Testing. *Journal of the Royal Statistical Society. Series B (Methodological)* **1995**, *57*, 289-300.
9. Huang, T.C.; Renuse, S.; Pinto, S.; Kumar, P.; Yang, Y.; Chaerkady, R.; Godsey, B.; Mendell, J.T.; Halushka, M.K.; Civin, C.I.; et al. Identification of miR-145 targets through an integrated omics analysis. *Molecular bioSystems* **2015**, *11*, 197-207, doi:10.1039/c4mb00585f.
10. Geekiyanage, H.; Chan, C. MicroRNA-137/181c regulates serine palmitoyltransferase and in turn amyloid beta, novel targets in sporadic Alzheimer's disease. *The Journal of neuroscience : the official journal of the Society for Neuroscience* **2011**, *31*, 14820-14830, doi:10.1523/JNEUROSCI.3883-11.2011.
11. Su, X.; Ye, Y.; Yang, Y.; Zhang, K.; Bai, W.; Chen, H.; Kang, E.; Kong, C.; He, X. The Effect of SPTLC2 on Promoting Neuronal Apoptosis is Alleviated by MiR-124-3p Through TLR4 Signalling Pathway. *Neurochemical research* **2019**, *44*, 2113-2122, doi:10.1007/s11064-019-02849-7.
12. Huang, S.; Lu, W.; Ge, D.; Meng, N.; Li, Y.; Su, L.; Zhang, S.; Zhang, Y.; Zhao, B.; Miao, J. A new microRNA signal pathway regulated by long noncoding RNA TGFB2-OT1 in autophagy and inflammation of vascular endothelial cells. *Autophagy* **2015**, *11*, 2172-2183, doi:10.1080/15548627.2015.1106663.
13. Fu, S.; Luan, T.; Jiang, C.; Huang, Y.; Li, N.; Wang, H.; Wang, J. miR-3622a promotes proliferation and invasion of bladder cancer cells by downregulating LASS2. *Gene* **2019**, *701*, 23-31, doi:10.1016/j.gene.2019.02.083.
14. Wang, H.; Zhang, W.; Zuo, Y.; Ding, M.; Ke, C.; Yan, R.; Zhan, H.; Liu, J.; Wang, J. miR-9 promotes cell proliferation and inhibits apoptosis by targeting LASS2 in bladder cancer. *Tumour biology : the journal of the International Society for Oncodevelopmental Biology and Medicine* **2015**, *36*, 9631-9640, doi:10.1007/s13277-015-3713-7.

15. Yu, B.; Zhou, S.; Wang, Y.; Qian, T.; Ding, G.; Ding, F.; Gu, X. miR-221 and miR-222 promote Schwann cell proliferation and migration by targeting LASS2 after sciatic nerve injury. *Journal of cell science* **2012**, *125*, 2675-2683, doi:10.1242/jcs.098996.
16. Su, B.; Han, H.; Ji, C.; Hu, W.; Yao, J.; Yang, J.; Fan, Y.; Li, J. MiR-21 promotes calcium oxalate-induced renal tubular cell injury by targeting PPARA. *American journal of physiology. Renal physiology* **2020**, *319*, F202-F214, doi:10.1152/ajprenal.00132.2020.
17. Cai, K.; Li, T.; Guo, L.; Guo, H.; Zhu, W.; Yan, L.; Li, F. Long non-coding RNA LINC00467 regulates hepatocellular carcinoma progression by modulating miR-9-5p/PPARA expression. *Open biology* **2019**, *9*, 190074, doi:10.1098/rsob.190074.
18. Xue, H.; Wang, B.; Xue, Y.S. LncRNA HOTAIR regulates the proliferation and apoptosis of vascular smooth muscle cells through targeting miRNA-130b-3p/PPARalpha axis. *European review for medical and pharmacological sciences* **2019**, *23*, 10989-10995, doi:10.26355/eurrev_201912_19804.
19. Chen, Z.; Luo, J.; Sun, S.; Cao, D.; Shi, H.; Loor, J.J. miR-148a and miR-17-5p synergistically regulate milk TAG synthesis via PPARGC1A and PPARA in goat mammary epithelial cells. *RNA biology* **2017**, *14*, 326-338, doi:10.1080/15476286.2016.1276149.
20. Liu, Y.; Xu, L.; Lu, B.; Zhao, M.; Li, L.; Sun, W.; Qiu, Z.; Zhang, B. LncRNA H19/microRNA-675/PPARalpha axis regulates liver cell injury and energy metabolism remodelling induced by hepatitis B X protein via Akt/mTOR signalling. *Molecular immunology* **2019**, *116*, 18-28, doi:10.1016/j.molimm.2019.09.006.
21. Hu, W.; Wang, X.; Ding, X.; Li, Y.; Zhang, X.; Xie, P.; Yang, J.; Wang, S. MicroRNA-141 represses HBV replication by targeting PPARA. *PloS one* **2012**, *7*, e34165, doi:10.1371/journal.pone.0034165.
22. Iliopoulos, D.; Malizos, K.N.; Oikonomou, P.; Tsezou, A. Integrative microRNA and proteomic approaches identify novel osteoarthritis genes and their collaborative metabolic and inflammatory networks. *PloS one* **2008**, *3*, e3740, doi:10.1371/journal.pone.0003740.
23. Zhang, S.; Yin, Z.; Dai, F.F.; Wang, H.; Zhou, M.J.; Yang, M.H.; Zhang, S.F.; Fu, Z.F.; Mei, Y.W.; Zang, M.X.; et al. miR-29a attenuates cardiac hypertrophy through inhibition of PPARdelta expression. *Journal of cellular physiology* **2019**, *234*, 13252-13262, doi:10.1002/jcp.27997.
24. Wang, M.Q.; Zhou, C.H.; Cong, S.; Han, D.X.; Wang, C.J.; Tian, Y.; Zhang, J.B.; Jiang, H.; Yuan, B. Lipopolysaccharide inhibits triglyceride synthesis in dairy cow mammary epithelial cells by upregulating miR-27a-3p, which targets the PPARG gene. *Journal of dairy science* **2021**, *104*, 989-1001, doi:10.3168/jds.2020-18270.
25. Chen, C.; Deng, Y.; Hu, X.; Ren, H.; Zhu, J.; Fu, S.; Xie, J.; Peng, Y. miR-128-3p regulates 3T3-L1 adipogenesis and lipolysis by targeting Pparg and Sertad2. *Journal of physiology and biochemistry* **2018**, *74*, 381-393, doi:10.1007/s13105-018-0625-1.
26. Yuan, Y.; Peng, W.; Liu, Y.; Xu, Z. Circulating miR-130 and its target PPAR-gamma may be potential biomarkers in patients of coronary artery disease with type 2 diabetes mellitus. *Molecular genetics & genomic medicine* **2019**, *7*, e909, doi:10.1002/mgg3.909.
27. Yang, G.; Zhou, L.; Xu, Q.; Meng, F.; Wan, Y.; Meng, X.; Wang, L.; Zhang, L. LncRNA KCNQ1OT1 inhibits the radiosensitivity and promotes the tumorigenesis of hepatocellular carcinoma via the miR-146a-5p/ACER3 axis. *Cell cycle* **2020**, *19*, 2519-2529, doi:10.1080/15384101.2020.1809259.
28. Yao, C.; Ruan, J.W.; Zhu, Y.R.; Liu, F.; Wu, H.M.; Zhang, Y.; Jiang, Q. The therapeutic value of the SphK1-targeting microRNA-3677 in human osteosarcoma cells. *Aging* **2020**, *12*, 5399-5410, doi:10.18632/aging.102961.
29. Wang, Z.; Qu, H.; Gong, W.; Liu, A. Up-regulation and tumor-promoting role of SPHK1 were attenuated by miR-330-3p in gastric cancer. *IUBMB life* **2018**, *70*, 1164-1176, doi:10.1002/iub.1934.

30. Chen, H.S.; Lu, A.Q.; Yang, P.Y.; Liang, J.; Wei, Y.; Shang, Y.W.; Li, Q. MicroRNA-28-5p regulates glioma cell proliferation, invasion and migration by targeting SphK1. *European review for medical and pharmacological sciences* **2019**, *23*, 6621-6628, doi:10.26355/eurrev_201908_18551.
31. Wang, D.; Bao, F.; Teng, Y.; Li, Q.; Li, J. MicroRNA-506-3p initiates mesenchymal-to-epithelial transition and suppresses autophagy in osteosarcoma cells by directly targeting SPHK1. *Bioscience, biotechnology, and biochemistry* **2019**, *83*, 836-844, doi:10.1080/09168451.2019.1569496.
32. Zhang, H.; Wang, Q.; Zhao, Q.; Di, W. MiR-124 inhibits the migration and invasion of ovarian cancer cells by targeting SphK1. *Journal of ovarian research* **2013**, *6*, 84, doi:10.1186/1757-2215-6-84.
33. Yu, H.; Duan, P.; Zhu, H.; Rao, D. miR-613 inhibits bladder cancer proliferation and migration through targeting SphK1. *American journal of translational research* **2017**, *9*, 1213-1221.
34. Liu, Z.; He, C.; Qu, Y.; Chen, X.; Zhu, H.; Xiang, B. MiR-659-3p regulates the progression of chronic myeloid leukemia by targeting SPHK1. *International journal of clinical and experimental pathology* **2018**, *11*, 2470-2478.
35. Xiao, G.; Wang, Q.; Li, B.; Wu, X.; Liao, H.; Ren, Y.; Ai, N. MicroRNA-338-3p Suppresses Proliferation of Human Liver Cancer Cells by Targeting SphK2. *Oncology research* **2018**, *26*, 1183-1189, doi:10.3727/096504018X15151495109394.
36. Bao, Y.; Chen, Z.; Guo, Y.; Feng, Y.; Li, Z.; Han, W.; Wang, J.; Zhao, W.; Jiao, Y.; Li, K.; et al. Tumor suppressor microRNA-27a in colorectal carcinogenesis and progression by targeting SGPP1 and Smad2. *PloS one* **2014**, *9*, e105991, doi:10.1371/journal.pone.0105991.
37. Huang, X.; Taeb, S.; Jahangiri, S.; Emmenegger, U.; Tran, E.; Bruce, J.; Mesci, A.; Korpela, E.; Vesprini, D.; Wong, C.S.; et al. miRNA-95 mediates radioresistance in tumors by targeting the sphingolipid phosphatase SGPP1. *Cancer research* **2013**, *73*, 6972-6986, doi:10.1158/0008-5472.CAN-13-1657.
38. Ruoming, W.; Zhen, Y.; Tengteng, Z.; Jisheng, H. Tumor suppressor microRNA-31 inhibits gastric carcinogenesis by targeting Smad4 and SGPP2. *Cancer gene therapy* **2015**, *22*, 564-572, doi:10.1038/cgt.2015.41.
39. Yang, W.; Wang, A.; Zhao, C.; Li, Q.; Pan, Z.; Han, X.; Zhang, C.; Wang, G.; Ji, C.; Wang, G.; et al. miR-125b Enhances IL-8 Production in Early-Onset Severe Preeclampsia by Targeting Sphingosine-1-Phosphate Lyase 1. *PloS one* **2016**, *11*, e0166940, doi:10.1371/journal.pone.0166940.
40. Zhou, P.; Huang, G.; Zhao, Y.; Zhong, D.; Xu, Z.; Zeng, Y.; Zhang, Y.; Li, S.; He, F. MicroRNA-363-mediated downregulation of S1PR1 suppresses the proliferation of hepatocellular carcinoma cells. *Cellular signalling* **2014**, *26*, 1347-1354, doi:10.1016/j.cellsig.2014.02.020.
41. Hu, D.; Sun, S.; Wang, Y. MicroRNA-455-5p exerts inhibitory effect in cervical carcinoma through targeting S1PR1 and blocking mTOR pathway. *Archives of gynecology and obstetrics* **2020**, *301*, 1307-1315, doi:10.1007/s00404-020-05536-z.
42. Miao, J.; Zhu, Y.; Xu, L.; Huang, X.; Zhou, X. miR181b5p inhibits trophoblast cell migration and invasion through targeting S1PR1 in multiple abnormal trophoblast invasionrelated events. *Molecular medicine reports* **2020**, *22*, 4442-4451, doi:10.3892/mmr.2020.11515.
43. Chen, A.; Wen, J.; Lu, C.; Lin, B.; Xian, S.; Huang, F.; Wu, Y.; Zeng, Z. Inhibition of miR1555p attenuates the valvular damage induced by rheumatic heart disease. *International journal of molecular medicine* **2020**, *45*, 429-440, doi:10.3892/ijmm.2019.4420.

44. Cheng, N.; Wang, G.H. miR-133b, a microRNA targeting S1PR1, suppresses nasopharyngeal carcinoma cell proliferation. *Experimental and therapeutic medicine* **2016**, *11*, 1469-1474, doi:10.3892/etm.2016.3043.
45. Guzzolino, E.; Chiavacci, E.; Ahuja, N.; Mariani, L.; Evangelista, M.; Ippolito, C.; Rizzo, M.; Garrity, D.; Cremisi, F.; Pitto, L. Post-transcriptional Modulation of Sphingosine-1-Phosphate Receptor 1 by miR-19a Affects Cardiovascular Development in Zebrafish. *Frontiers in cell and developmental biology* **2018**, *6*, 58, doi:10.3389/fcell.2018.00058.
46. Wu, H.X.; Wang, G.M.; Lu, X.; Zhang, L. miR-542-3p targets sphingosine-1-phosphate receptor 1 and regulates cell proliferation and invasion of breast cancer cells. *European review for medical and pharmacological sciences* **2017**, *21*, 108-114.
47. Li, Q.; Pan, Z.; Wang, X.; Gao, Z.; Ren, C.; Yang, W. miR-125b-1-3p inhibits trophoblast cell invasion by targeting sphingosine-1-phosphate receptor 1 in preeclampsia. *Biochemical and biophysical research communications* **2014**, *453*, 57-63, doi:10.1016/j.bbrc.2014.09.059.
48. Fan, J.L.; Zhang, L.; Bo, X.H. MiR-126 on mice with coronary artery disease by targeting S1PR2. *European review for medical and pharmacological sciences* **2020**, *24*, 893-904, doi:10.26355/eurrev_202001_20074.
49. Wan, Y.; Jin, H.J.; Zhu, Y.Y.; Fang, Z.; Mao, L.; He, Q.; Xia, Y.P.; Li, M.; Li, Y.; Chen, X.; et al. MicroRNA-149-5p regulates blood-brain barrier permeability after transient middle cerebral artery occlusion in rats by targeting S1PR2 of pericytes. *FASEB journal : official publication of the Federation of American Societies for Experimental Biology* **2018**, *32*, 3133-3148, doi:10.1096/fj.201701121R.
50. Gong, L.; Wu, X.; Li, X.; Ni, X.; Gu, W.; Wang, X.; Ji, H.; Hu, L.; Zhu, L. S1PR3 deficiency alleviates radiation-induced pulmonary fibrosis through the regulation of epithelial-mesenchymal transition by targeting miR-495-3p. *Journal of cellular physiology* **2020**, *235*, 2310-2324, doi:10.1002/jcp.29138.
51. Zhai, L.; Wu, R.; Han, W.; Zhang, Y.; Zhu, D. miR-127 enhances myogenic cell differentiation by targeting S1PR3. *Cell death & disease* **2017**, *8*, e2707, doi:10.1038/cddis.2017.128.
52. Tian, J.; Fan, J.; Xu, J.; Ren, T.; Guo, H.; Zhou, L. circ-FNTA accelerates proliferation and invasion of bladder cancer. *Oncology letters* **2020**, *19*, 1017-1023, doi:10.3892/ol.2019.11150.
53. Kukreti, H.; Amuthavalli, K. MicroRNA-34a causes ceramide accumulation and effects insulin signaling pathway by targeting ceramide kinase (CERK) in aging skeletal muscle. *Journal of cellular biochemistry* **2020**, *121*, 3070-3089, doi:10.1002/jcb.29312.
54. Umeh-Garcia, M.; Simion, C.; Ho, P.Y.; Batra, N.; Berg, A.L.; Carraway, K.L.; Yu, A.; Sweeney, C. A Novel Bioengineered miR-127 Prodrug Suppresses the Growth and Metastatic Potential of Triple-Negative Breast Cancer Cells. *Cancer research* **2020**, *80*, 418-429, doi:10.1158/0008-5472.CAN-19-0656.
55. Lv, T.; Song, K.; Zhang, L.; Li, W.; Chen, Y.; Diao, Y.; Yao, Q.; Liu, P. miRNA-34a decreases ovarian cancer cell proliferation and chemoresistance by targeting HDAC1. *Biochemistry and cell biology = Biochimie et biologie cellulaire* **2018**, *96*, 663-671, doi:10.1139/bcb-2018-0031.
56. Abdelfattah, N.; Rajamanickam, S.; Panneerdoss, S.; Timilsina, S.; Yadav, P.; Onyeagucha, B.C.; Garcia, M.; Vadlamudi, R.; Chen, Y.; Brenner, A.; et al. MiR-584-5p potentiates vincristine and radiation response by inducing spindle defects and DNA damage in medulloblastoma. *Nature communications* **2018**, *9*, 4541, doi:10.1038/s41467-018-06808-8.
57. Liu, T.; Hou, L.; Zhao, Y.; Huang, Y. Epigenetic silencing of HDAC1 by miR-449a upregulates Runx2 and promotes osteoblast differentiation. *International journal of molecular medicine* **2015**, *35*, 238-246, doi:10.3892/ijmm.2014.2004.

58. Lu, J.; Zhou, Z.; Sun, B.; Han, B.; Fu, Q.; Han, Y.; Yuan, W.; Xu, Z.; Chen, A. MiR-520d-5p modulates chondrogenesis and chondrocyte metabolism through targeting HDAC1. *Aging* **2020**, *12*, 18545-18560, doi:10.18632/aging.103831.
59. Tang, W.; Zhou, W.; Xiang, L.; Wu, X.; Zhang, P.; Wang, J.; Liu, G.; Zhang, W.; Peng, Y.; Huang, X.; et al. The p300/YY1/miR-500a-5p/HDAC2 signalling axis regulates cell proliferation in human colorectal cancer. *Nature communications* **2019**, *10*, 663, doi:10.1038/s41467-018-08225-3.
60. Dai, W.; Dai, J.L.; Tang, M.H.; Ye, M.S.; Fang, S. lncRNA-SNHG15 accelerates the development of hepatocellular carcinoma by targeting miR-490-3p/ histone deacetylase 2 axis. *World journal of gastroenterology* **2019**, *25*, 5789-5799, doi:10.3748/wjg.v25.i38.5789.
61. Mao, G.; Zhang, Z.; Huang, Z.; Chen, W.; Huang, G.; Meng, F.; Zhang, Z.; Kang, Y. MicroRNA-92a-3p regulates the expression of cartilage-specific genes by directly targeting histone deacetylase 2 in chondrogenesis and degradation. *Osteoarthritis and cartilage* **2017**, *25*, 521-532, doi:10.1016/j.joca.2016.11.006.
